# Hyperglycemia Negatively Affects IPSC-Derived Myoblast Proliferation and Skeletal Muscle Regeneration and Function

**DOI:** 10.3390/cells11223674

**Published:** 2022-11-18

**Authors:** Agnes Badu-Mensah, Paola Valinski, Hemant Parsaud, James J. Hickman, Xiufang Guo

**Affiliations:** 1 NanoScience Technology Center, University of Central Florida, 12424 Research Parkway, Suite 400, Orlando, FL 32826, USA; 2 Burnett School of Biomedical Sciences, College of Medicine, University of Central Florida, Orlando, FL 32816, USA

**Keywords:** hyperglycemia, skeletal muscle, myoblast, iPSC, in vitro

## Abstract

Diabetic myopathy is a co-morbidity diagnosed in most diabetes mellitus patients, yet its pathogenesis is still understudied, which hinders the development of effective therapies. This project aimed to investigate the effect of hyperglycemia on human myoblast physiology, devoid of other complicating factors, by utilizing human myoblasts derived from induced pluripotent stem cells (iPSCs), in a defined in vitro system. IPSC-derived myoblasts were expanded under three glucose conditions: low (5 mM), medium (17.5 mM) or high (25 mM). While hyperglycemic myoblasts demonstrated upregulation of Glut4 relative to the euglycemic control, myoblast proliferation demonstrated a glucose dose-dependent impedance. Further cellular analysis revealed a retarded cell cycle progression trapped at the S phase and G2/M phase and an impaired mitochondrial function in hyperglycemic myoblasts. Terminal differentiation of these hyperglycemic myoblasts resulted in significantly hypertrophic and highly branched myotubes with disturbed myosin heavy chain arrangement. Lastly, functional assessment of these myofibers derived from hyperglycemic myoblasts demonstrated comparatively increased fatigability. Collectively, the hyperglycemic myoblasts demonstrated deficient muscle regeneration capability and functionality, which falls in line with the sarcopenia symptoms observed in diabetic myopathy patients. This human-based iPSC-derived skeletal muscle hyperglycemic model provides a valuable platform for mechanistic investigation of diabetic myopathy and therapeutic development.

## 1. Introduction

Diabetic myopathy is a co-morbidity diagnosed in about 88% of all diabetes mellitus (DM) patients [1]. It is characterized by the progressive decline in skeletal muscle (SKM) mass, metabolism and function [2]. Compared to other common diabetic co-morbidities, diabetic myopathy is relatively understudied, but is believed to directly influence the rate of co-morbidity development, based on the fact that skeletal muscle is the largest site for glucose uptake and thus a vital organ for glucose homeostasis and overall metabolism regulation [3]. If left untreated, diabetic myopathy can negatively affect the overall quality of life and survivability of patients as a result of its negative impact on many metabolic processes, including glucose and lipid metabolism as well as insulin secretion [2]. Thus, understanding the cause of diabetic myopathy onset and progression is important in order to effectively manage the condition and develop better therapeutics. However, the factors and cellular mechanisms triggering diabetic myopathy are still poorly understood.

Under diabetic conditions, especially poorly managed diabetic states, there may be a multitude of diabetic-related factors and processes that can potentially influence muscle physiology, such as hyperglycemia, dyslipidemia [4] and chronic low-grade inflammation [5]. Among the many factors, hyperglycemia is commonly shared among all types of Diabetes Mellitus [2,6]. Hyperglycemia is also observed in other conditions, such as gestational diabetes, which occurs in 4% of pregnancies. Additionally, aging-related change in glucose metabolism is a known risk factor of hyperglycemia [7]. Therefore, it is important to elucidate the effect of hyperglycemia on muscle physiology, maintenance and regeneration. 

Numerous studies based on clinical data or animal models have indicated that hyperglycemia has been implicated in altering skeletal muscle morphology, metabolism and function [8,9]. Some studies have speculated that hyperglycemia directly impacts SKM regeneration, maintenance and functional capacity [2,10]. Accumulative evidence has also indicated that an uncontrolled diabetic environment would negatively affect the muscle progenitor cell population (particularly the muscle satellite cell population), and as such, likely contributes to the declining skeletal muscle health observed in diabetes mellitus [6,11]. However, this fact remains inconclusive due to the complexity of the DM syndrome, and the limitations of animal models [2,12,13]. Therefore, a defined human-based in vitro system is needed to ascertain the effect of hyperglycemia on muscle physiology. A protocol for differentiating human skeletal myofibers from induced pluripotent stem cells (iPSCs) has been reported [14], and a system for analyzing the cellular pathology/physiology and function has been well established in our group [15]. This iPSC-derived in vitro system will be utilized for this study.

While studies concerning the link between changes to skeletal muscle metabolic health following diabetes mellitus onset (particularly Type 2 diabetes mellitus) have been the primary focus [16], and significant progress has been reported recently [17], few have examined the negative impact of diabetes mellitus on the growth and reparative capacities of skeletal muscle that often coincides with disease development. Understanding the effect of hyperglycemia on muscle growth and reparative capacities would shed light on the mechanisms of deficient muscle development in Type I diabetes mellitus (T1DM) and impaired muscle maintenance in Type II diabetes (T2DM). By utilizing human myoblasts derived from induced pluripotent stem cells (iPSCs) in a defined in vitro system devoid of other complicating factors, this study will investigate the specific effect of hyperglycemia exposure at the myoblast stage on human myoblast proliferation, metabolism, myogenic capability, and the structure and function of generated myotubes.

## 2. Materials and Methods

### 2.1. Experimental Design

**Figure 1:**
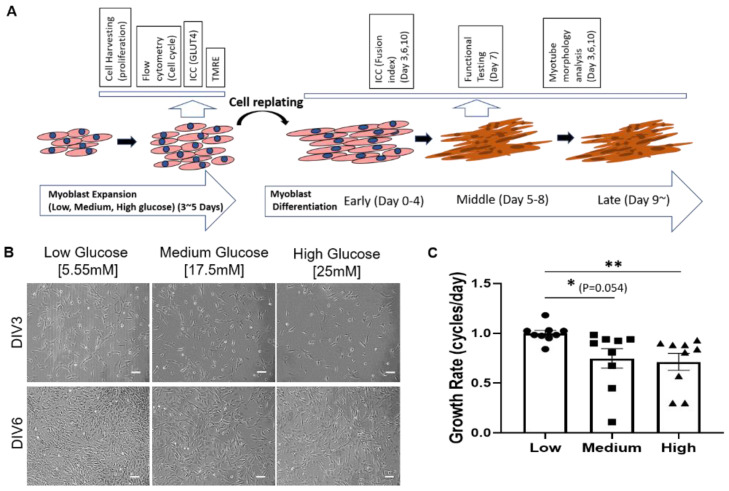
Effect of hyperglycemia on iPSC-derived myoblast proliferation. (**A**) Schematic illustration of iPSC-derived myoblast culturing and differentiation timeline, and the experiments at different stages. (**B**) Phase images of iPSC-derived myoblasts in 5mM, 17.5mM and 25mM of glucose at days 3 (Days in vitro or DIV 3) and 6 (DIV 6) of proliferation. (**C**) Graph showing primary myoblasts proliferation rate in varying glucose concentration. Scale bar = 100 µm. Error bar: SEM. *p*-value *P* ≤ 0.1, *; *P* ≤ 0.05, **; *P* ≤ 0.01, ***. *n* = 5 in 2 experiments. Statistics was performed utilizing One-way ANOVA with post-hoc Tukey HSD Test by comparing between each hyperglycemia group with the euglycemic control group.

Glucose supply is an important factor for muscle physiology. Normal plasma glucose concentration is around 5.55 mM (1000 mg/L). In cell culture, different glucose concentrations have been utilized in commercial media: 5.55 mM (1g/L), 17.5 mM (3.15 g/L) and 25 mM (4.5 g/L) are representative. To investigate the effect of hyperglycemia for both in vivo and in vitro environments, similar glucose concentrations were chosen in this study, with 5.55 mM as a simulator of the normal glucose condition, and 17.5 mM and 25 mM as medium and high glucose conditions, respectively. The effect of hyperglycemia on myogenesis was investigated at the myoblast stage by evaluating myoblast proliferation, mitochondria function (TMRE assay), Glut4 expression, and at the early stage of myotube formation for their potential for myotube formation (fusion index). The effect of hyperglycemia on myofiber structure and function was investigated by examining myotube morphology and contractile properties under electrical stimulation. The overall experimental scheme is illustrated in Figure 1A. 

### 2.2. Surface Modification

Acid-washed coverslips were prepared by soaking them in 3M hydrochloric acid and placing them on a shaker overnight. The coverslips were then rinsed thrice with deionized water and soaked in 100% ethanol for storage.

### 2.3. Myoblast Derivation

IPSCs derived from a healthy subject were purchased from the Coriell Institute and were differentiated as described in a previous publication from our laboratory [15,18]. This protocol was adapted from one published by Chal et al. and slightly modified for our perusal [14,15]. Briefly, iPSCs were plated on a Matrigel-coated (Cat# 354230 Corning Life Sciences, Durham, NC, USA) surface in mTESR and cultured until reaching 15–30% confluence, at which point the differentiation protocol was initiated. This small molecule-directed differentiation involved the parallel activation of Wnt and the inhibition of BMP pathways by utilizing CHIR-99021 and LDN-193189 [14]. At the end of the differentiation, the now dense culture was replated at a lower density for morphological assessment and to allow the enrichment and proliferation of the myogenic cells. The iPSC-myoblasts were characterized by immunostaining with the markers, PAX7 and MyoD, as described in a previous publication [15]. Confirmed myoblasts were expanded until passage 3 in Myocult medium (MyoCult™-SF Expansion 10X Supplement (Human), cat# 05982 and DMEM with 1000 mg/L D-Glucose, cat# 36253, StemCell Technologies, Vancouver, Canada), after which they were harvested and cryopreserved in 10% DMSO for subsequent experiments. Primary myoblasts were purchased from Lonza and passaged once in Myocult medium before use in experiments. 

### 2.4. Hyperglycemic Proliferation Medium Preparation and Treatment Experiment

Myocult medium comes with 1000 mg/mL (5.55 mM) glucose and was used as the low glucose condition. Supplementing this medium with 107.55 mg and 175.05 mg of dextrose (D-glucose) made 50 mls of medium glucose (17.5 mM) and high glucose (25 mM) Myocult media, respectively. IPSC-derived myoblasts and primary myoblasts were plated at 50 cells per mm^2^ on collagen I-coated surfaces in either low (5.55 mM), medium (17.5 mM) or high (25 mM) in triplicate and allowed to proliferate for a few days. Myoblasts were harvested using TrypLE (cat# 12605010, ThermoFisher, Waltham, MA, USA) for each condition and counted. Using the cell count results, the growth rates (i.e., cycles per day) of both primary and iPSC-derived myoblasts in each D-glucose condition were calculated using the Equation (1).

(1) $$C\;\left(cycles\;per\;day\right)=\frac{Ln\left(\frac{End\;cell\;count\;after\;harvesting\;}{Initial\;cell\;count\;at\;plating}\right)}{Days\;in\;growth}.$$

### 2.5. Myoblast Cell Cycle Assay

DNA content-based cell cycle analysis was performed using the SYTOX AADvanced^TM^ Dead Cell Stain Kits (cat# S10274, Thermofisher) with RNase A (cat# 12091-039, Thermofisher) for DNA-specific signals. As described above, iPSC-derived and primary myoblasts were plated in collagen-coated T-75 flasks in Myocult medium containing 5.55 mM, 17.5 mM or 25 mM glucose, and allowed to proliferate for 7 days before harvesting. The cell cycle assay was performed strictly per the manufacturer’s instruction and ran on CytoFLEX LX (Beckman Coulter, Brea, CA, USA) flow cytometer. Resulting data were analyzed using CytExpert software, version 2.1 (Beckman Coulter). 

### 2.6. Hyperglycemia Myoblast Differentiation

As in Figure 1A, after prior expansion in either 5.55 mM, 17.5 mM, or 25 mM proliferation medium, iPSC myoblasts were replated at 200 cells/mm^2^ on collagen-coated acid washed coverslips. The myoblasts were allowed to proliferate to about 70% confluence in the respective expansion media for 3 days, after which they were switched to priming medium (DK-HI) for 48 hours before being switched to differentiation (N2) medium. The DK-HI medium and N2 medium were prepared as described in [14,15]. Specifically, DK-HI consists of DMEM/F12 (cat# 21041-025, ThermoFisher Scientific), 1% Insulin Transferrin Selenium (ITS) (cat# 41400045, ThermoFisher Scientific), 1% N2 supplement (cat# 17502048, ThermoFisher Scientific), 1% L-glutamine 20 mM (cat# A2916801, ThermoFisher Scientific), 100 µM dexamethasone (cat# D4902, Sigma, MO, USA). N2 medium consists of DMEM/F12 (cat# 21041-025, ThermoFisher Scientific), 1% Insulin Transferrin Selenium (ITS) (cat# 41400045, ThermoFisher Scientific), 1% N2 supplement (cat# 17502048, ThermoFisher Scientific), 1% L-glutamine 20 mM (cat# A2916801 ThermoFisher Scientific), 100 nM dexamethasone (cat# D4902, Sigma, Saint Louis, MO, USA). Priming and differentiation parameters were the same for all three culture conditions. Differentiation medium was refreshed every other day until cultures were ready for immunocytochemical or functional analysis.

### 2.7. Fusion Index and Branched Myotube Quantification

Per the glucose condition, coverslips were fixed on days 3, 6 and 10. The myotubes were stained with anti-MyHC and 4′,6-diamidino-2-phenylindole (DAPI) and imaged at 20X magnification using confocal microscopy. Five random images were collected per coverslip. The images were quantified using ImageJ software version 1.53 (NIH). Fusion Index was calculated as the number of nuclei within myotubes normalized to the total number of nuclei (Equation (2)). Percent of Branched Myotubes was calculated as the number of branched points normalized to the total number of myotubes in the image (Equation (3)).

(2) $$Fusion\;Index\;=\;\left(\frac{number\;of\;DAPI\;signals\;within\;\;myotubes}{total\;number\;of\;DAPI\;signals}\right).$$

(3) $$Percent\;of\;Branched\;Myotubes\;=\;\left(\frac{number\;of\;branched\;points}{total\;number\;of\;myotubes}\right).$$

### 2.8. Mitochondrial Function Assessment

iPSC-derived myoblasts were plated in Myocult medium at the three glycemic levels at 50 cells/mm^2^ on Collagen I-coated 18 mm glass coverslips. The myoblasts were allowed to proliferate in correspondent media until day 5, when they were incubated in a 500 nM tetramethylrhodamine (TMRE) (Thermofisher Scientific, Cat# T669) solution for 30 min and rinsed twice with 1× PBS. Fluorescence images of the myoblasts in each condition were taken with a Zeiss spinning disk confocal microscope (Axioskop 2 Mot Plus, Carl Zeiss, Oberkochen, Germany) to assess the TMRE intensity. Average fluorescence intensity per image was measured via ImageJ software (version 1.8.0). 

### 2.9. Immunocytochemistry

Cultures were fixed with a 4% PFA solution for 15 minutes, rinsed 3X with 1× PBS and permeabilized with a 0.1% triton solution. Cells were then blocked for an hour with donkey serum blocking buffer (2.5 mL Donkey Serum + 2.5 mL BSA + 40 mL sterile water + 5mLs PBS 10×) after a 15-min permeabilization period with 0.1% triton in PBS. Primary antibodies for MyoD (Cat# LS-C263888, LS Bio, WA, USA), Pax7 (Cat# PAS-117P, Invitrogen, MA, USA) and Ki67 (Cat# ab238020, Abcam, Cambridge, UK), MyHC (cat# A4.1025-s, DSHB, IA, USA) were added to the cultures and incubated overnight at 4 °C. The secondary antibodies (Thermofisher Scientific) were added the next day at a 1:250 dilution. Coverslips were rinsed 3X in 1× PBS and mounted on glass slides with Prolong gold mounting medium with DAPI (cat# P36931, ThermoFisher). Images were taken with an Axioscope spinning disk confocal microscope (Carl Zeiss) linked to XCite 120 Fluorescence Illumination system beam with laser scanning software, Volocity version 6.3.0 (Perkin Elmer, MA, USA).

### 2.10. Functional Assessment 

Myotubes on coverslips from all three culture conditions at 7 days in differentiation were assembled into acrylic housings placed on a motion capture-based rig equipped with a heated stage (ThorLabs TC200), an upright phase-contrast microscope (Zeiss Hal 100) and a high-speed acquisition digital camera (Hamamatsu model C8484-05G). Myotubes were stimulated for contraction at 0.3, 0.5, 1.0, 2.0 and 4.0 Hz via broad field stimulations for 15 s. Myotube contractions were captured using the camera from a selected region of interest (ROI). This procedure is described in detail by Santhanam et al. [19]. The myofiber contraction Fatigue Index was calculated using this formula as described in [18] (Equation (4)):

(4) $$Fatigue\;Index\;=1-\left(\frac{Area\;Under\;Curve}{Peak\;Force\;*\;Time}\right).$$

### 2.11. Statistics

All the statistics were performed utilizing One-way ANOVA with post-hoc Turkey HSD Test. Comparisons were made between each hyperglycemia group with the euglycemic control group, and between the two hyperglycemia conditions if relevant.

## 3. Results

### 3.1. High D-Glucose Levels Impede Myoblast Proliferation

The timeline from myoblast to functional myofibers and the time points for conducting correspondent experiments are illustrated in Figure 1A. To study the effect of hyperglycemia on SKM morphology and function, iPSC–derived myoblasts were plated at 50 cells/mm^2^ on collagen-coated polystyrene plates in proliferation medium with 1000 mg/L (5.55 mM) low, 3151 mg/L (17.5 mM) medium or 4501 mg/L (25 mM) high D-glucose and cultured over time. The first noticeable difference among different glucose conditions was a reduced increase of cell density with increasing D-glucose levels (Figure 1B). To quantify this observation, the proliferation rate in each condition was quantified after 7 days of myoblast expansion. Growth rate assessment showed a dose-dependent decrease in myoblast proliferation in hyperglycemic (i.e., 17.5 mM and 25 mM) cultures compared to euglycemic (i.e., 5 mM) cultures (Figure 1C). This experiment was repeated with primary myoblasts and resulted in the same outcome (Figure S1). Altogether, the results indicate that hyperglycemia negatively affects myoblast proliferation.

### 3.2. Hyperglycemia Disturbs Myoblast Myogenesis

**Figure 2:**
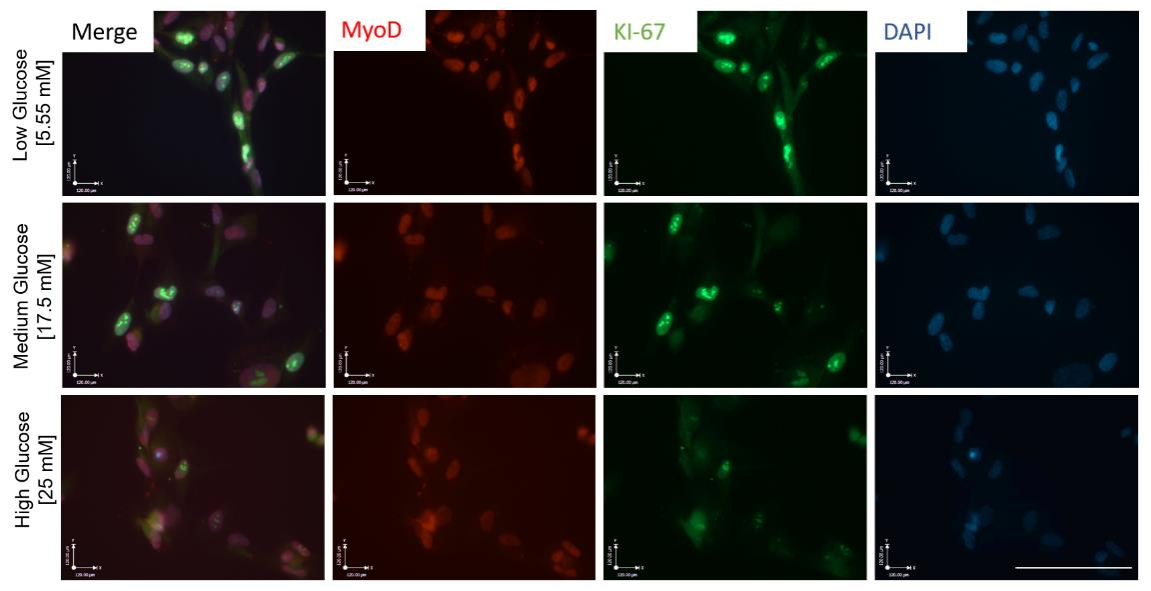
ICC for MyoD and KI-67 expression in iPSC-derived myoblasts cultured in medium of the three varying glucose concentrations, 5 days in vitro. Scale bar = 50 µm.

**Figure 3:**
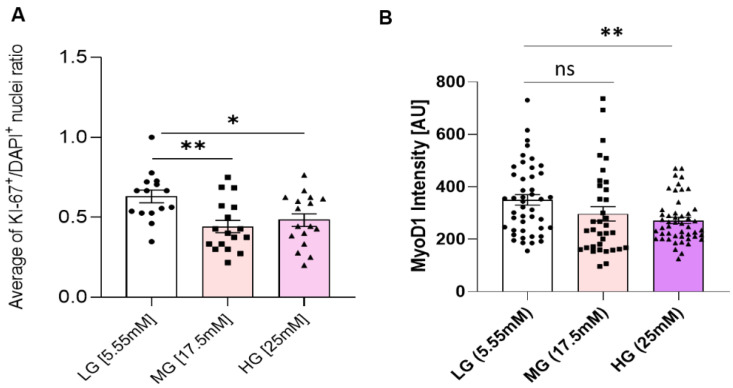
Proliferation and Myogenicity assessment. (**A**) Ratio of KI-67+/DAPI+ nuclei in iPSC-derived myoblasts cultured in euglycemic (5.55 mM) compared to hyperglycemic (17.5 mM, 25 mM) conditions. (**B**) Mean MyoD expression intensity quantification in euglycemic (5.55 mM) versus hyperglycemic (17.5 mM, 25 mM) myoblasts. Error bar: SEM. *P* ≤ 0.1, *; *P* ≤ 0.05, **; *P* ≤ 0.01, ***. A total of 15 imaging areas (covering more than 200 cells) from 2 experiments for each glucose condition were analyzed. Statistics was performed utilizing One-way ANOVA with post-hoc Tukey HSD Test.

To understand how the observed proliferation impedance occurs in hyperglycemic conditions, myoblasts expanded in euglycemic (5.55 mM) and hyperglycemic (17.5 mM and 25 mM) medium were fixed and stained for a proliferation marker Ki-67, and a proliferative myoblast marker MyoD (Figure 2). Quantification results showed a significantly lower ratio of Ki-67+/DAPI+ cells in hyperglycemic cultures compared to euglycemic controls (Figure 3A). Contrarily, there was no significant difference of MyoD+/DAPI+ cells among the various glycemic conditions, which was over 0.95, consistent with our previous publications [15,20]. However, MyoD signal intensity was found to be lower in hyperglycemic cultures relative to the control (Figure 3B). These findings demonstrate that hyperglycemia may directly interfere with myoblast proliferation. 

### 3.3. Hyperglycemia Retards Myoblast Cell Cycle Transversal

**Figure 4:**
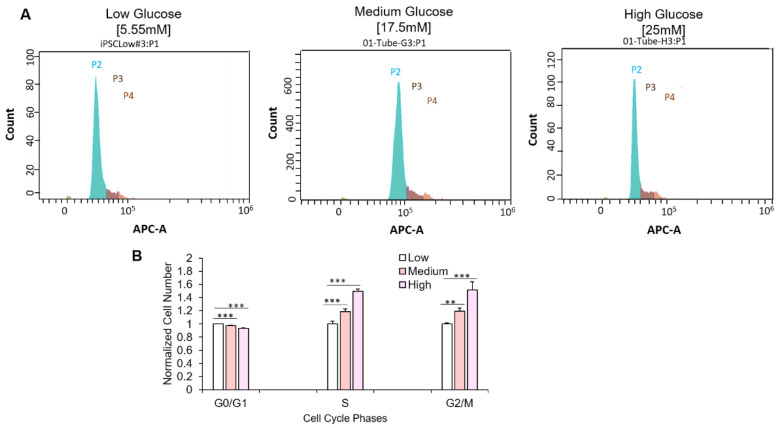
Effect of hyperglycemia on cell cycle progression. (**A**) Pictograph showing iPSC-derived -myoblast distribution among the various cell cycle phases in each glucose concentration (P2, green: G0/G1 phase; P3, dark brown, S phase; P4, light brown, G2/M phase). (**B**) Quantification of iPSC-myoblasts at the various phases of cell cycle in 5.55 mM, 17.5 mM and 25 mM D-glucose conditions. Data was normalized to the cell count of each phase at euglycemic condition. Error bars = SEM. *P* ≤ 0.1, *; *P* ≤ 0.05, **; *P* ≤ 0.01, ***. *n* = 10. Statistics was performed utilizing One-way ANOVA with post-hoc Tukey HSD Test.

Ki-67 is vital for cell cycle progression and is reported to be highly upregulated at the G1 phase but quickly degrades upon cell cycle arrest [21]. Having observed altered Ki-67 expression in myoblasts cultured in hyperglycemic conditions, cell cycle analysis was performed to assess the distribution of myoblasts among the various phases of the cell cycle. Myoblasts were cultured at the three glucose conditions as previously described and subsequently harvested for cell cycle assessment via flow cytometry. Compared to the euglycemic condition, a significantly increased number of myoblasts in the hyperglycemic conditions (medium and high D-glucose) were found in the S and G2/M phases (Figure 4). These experiments were also performed with primary myoblasts and yielded similar results (Figure S2). In summary, the results indicate that hyperglycemia affects myoblast cell cycle transversal from S and G2/M phases. 

### 3.4. Hyperglycemia Results in Dose-Dependent Increases in Glut4 Expression 

**Figure 5:**
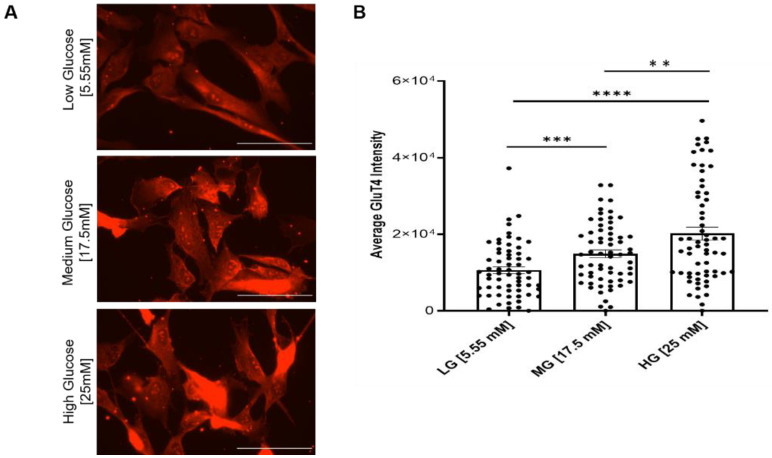
Analysis of Glut4 expression in myoblasts grown under different glucose conditions. (**A**) Immunocytochemical staining of Glut4 on euglycemic (5.55 mM) versus hyperglycemic (17.5 mM, 25 mM) iPSC-derived myoblasts. (**B**) Quantification of average Glut4 intensities at various glucose concentrations. *n* = 60 cells for each condition from 2 batches of experiment. Error bars = SEM. *P* ≤ 0.1, *; *P* ≤ 0.05, **; *P* ≤ 0.01, ***, *P* ≤ 0.001, ****. Statistics was performed utilizing One-way ANOVA with post-hoc Tukey HSD Test.

The decreased myoblast proliferation and impaired cell cycle progression suggested altered D-glucose uptake capability and/or mitochondrial dysfunction. Glucose transporter type 4 (Glut4) is an insulin-regulated glucose transporter found primarily in adipose tissues and striated muscle (skeletal and cardiac). As a membrane protein, Glut4 permits the facilitated diffusion of circulating glucose down its concentration gradient into muscle and fat cells. Once within the cells, glucose is rapidly phosphorylated and enters glycolysis or is polymerized into glycogen. To detect any change in the glucose uptake capability of the myoblasts under different glucose conditions, Glut4 expression was assayed by immunocytochemistry across all culture conditions. The results indicated significant increases in Glut4 signal intensity in the hyperglycemic (17.5 and 25 mM) cultures relative to the euglycemic (5.55 mM) control (Figure 5), and higher intensity in 25 mM than in 17.5 mM, suggesting a dose-dependent upregulation of Glut4 in response to increasing glucose levels (Figure 5).

### 3.5. Hyperglycemia Negatively Affects Mitochondrial Function of iPSC Myoblast

**Figure 6:**
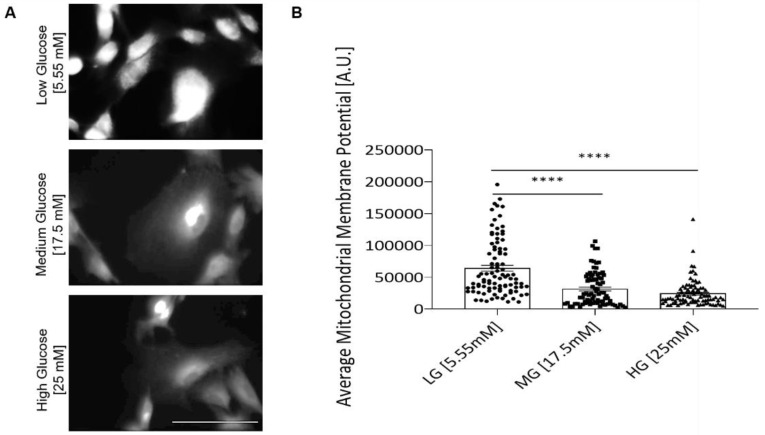
TMRE analysis of myoblasts grown under different glucose conditions. (**A**) Representative confocal images of TMRE staining in each D-glucose condition. (**B**) TMRE staining quantification for inner mitochondrial membrane potential of iPSC-derived myoblasts in euglycemic versus hyperglycemic conditions. Four coverslips from two experiments were quantified for each glucose condition. Error bars = SEM. *P* ≤ 0.1, *; *P* ≤ 0.05, **; *P* ≤ 0.01, ***, *P* ≤ 0.001, ****. Statistics was performed utilizing One-way ANOVA with post-hoc Tukey HSD Test.

To detect whether the cell’s mitochondria function was affected by hyperglycemia, the mitochondria inner membrane potential of the myoblasts was analyzed by utilizing a TMRE assay. TMRE analysis indicated a significant decline in the inner membrane potential in hyperglycemic myoblast mitochondria (Figure 6), suggesting the mitochondria’s ability to generate ATP was reduced. The experimental outcome suggests that deficient mitochondria function or inefficient ATP production may be a cause of compromised myoblast proliferation and cell cycle transversal.

### 3.6. Myoblasts Expanded under Hyperglycemic Conditions Demonstrated Accelerated Myotube Differentiation Profile

**Figure 7:**
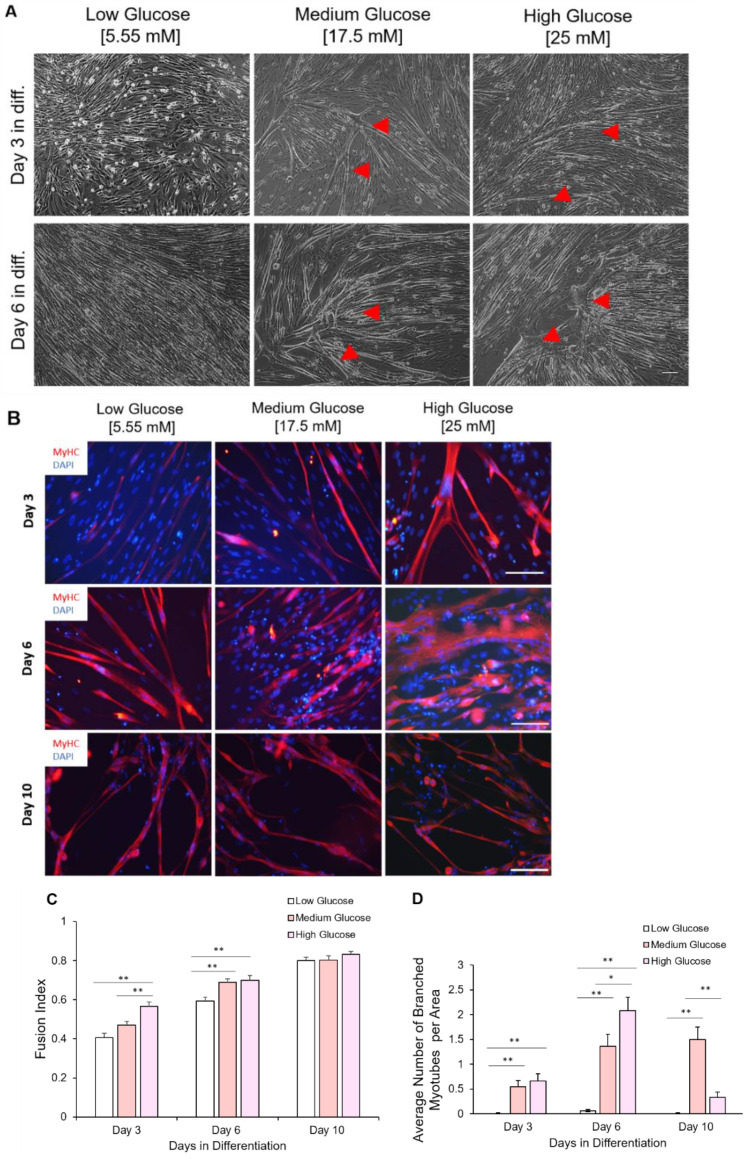
Chronological morphological and fusion index analysis of myotubes differentiated from iPSC-myoblasts expanded in the three D-glucose conditions. (**A**) Phase contrast imaging of myotubes at days 3 and 6 of differentiation. Myoblasts expanded under high glucose conditions tended to hyper-fuse and form branched myotubes (indicated by red arrows) after differentiation initiation. Scale bar = 100 µm. Red arrows pointing to branched hypertrophic myotubes. (**B**) ICC images of myotubes differentiated from iPSC-derived myoblasts previously cultured in the three D-glucose concentrations at days 3, 6 and 10 of differentiation. Myoblasts expanded under high glucose conditions tended to hyper-fuse and form branched myotubes after differentiation initiation. Those branched myotubes tended to detach sooner and few were left at day 10. Scale bar = 100 µm. (**C**) Quantification of Fusion Index of myotubes differentiated from iPSC-derived myoblasts previously proliferated in 5.55, 17.5 and 25 mM D-glucose at 3, 6 and 10 days of differentiation. (**D**) Quantification of branched myotubes per image area. Error bars = SEM. *P* ≤ 0.1, *; *P* ≤ 0.05, **; *P* ≤ 0.01, ***. *n* = 6. Statistics was performed utilizing One-way ANOVA with post-hoc Tukey HSD Test.

The initial exposure of myoblasts to different glycemic conditions also affects their terminal differentiation into myotubes. Myoblasts, having been expanded in different glycemic conditions, were replated at 200 cells/mm^2^ and subjected to the same myotube differentiation conditions. Myoblasts previously exposed to hyperglycemic (17.5 mM and 25 mM) conditions fused earlier compared to those expanded in euglycemic conditions for both iPSC-myoblast (Figure 7A) and primary myoblasts (Figure S3). Signs of hyper-fusion were evident in those myoblasts previously exposed to 17.5 mM and 25 mM D-glucose cultures as early as day 1 in differentiation. Fully formed myotubes were apparent in hyperglycemic cultures by day three in differentiation compared to their euglycemic counterparts (Figure 7B). The fusion index under medium and high glucose conditions was consistently higher than for the low glucose conditions at days 3 and 6 after initiation of terminal differentiation, while demonstrating a dose-dependent accelerated fusion at day 3. Conversely, fusion in myoblasts expanded under euglycemic conditions was steady and reached its peak by day 10 of differentiation (Figure 7B,C).

### 3.7. Myotubes Differentiated from Hyperglycemic Myoblasts Displayed Branched Morphology

Further examination of most myotubes differentiated from hyperglycemic myoblasts revealed a branched morphology, especially among the hyperfused myotubes, which was observed in both iPSC-myoblasts (Figure 7A,B) and primary myoblasts (S3). Branched hyperfused myotubes were observed in hyperglycemic cultures as early as day 3 in differentiation, reached a peak around day 6, but had mostly disappeared by day 10 in differentiation (especially in the 25 mM condition), presumably due to rupture caused by eccentric contraction stress (Figure 7D). Additionally, these branched, hyperfused myotubes demonstrated highly disorganized Myosin Heavy Chain (MyHC) that were most elaborate by day 6 in differentiation (Figure 7B). Similar hyper-fusion and formation of branched myotubes were also observed in the experiments utilizing human primary myoblasts (Figure S3). These results demonstrated that exposure of myoblasts to hyperglycemic conditions during the proliferative stage can have lasting effects that alter the eventual fusion and morphology of the myotubes.

### 3.8. Exposure of Myoblasts to Hyperglycemic Conditions during Proliferation Affected Myofiber Function

**Figure 8:**
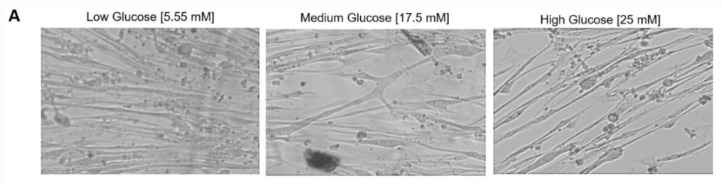

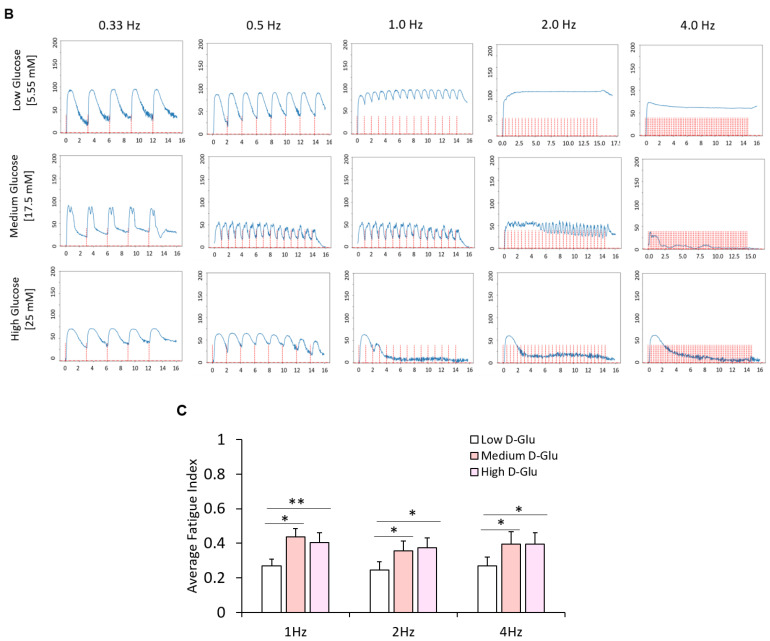
Functional characterization of myotubes differentiated from iPSC-derived myoblasts previously cultured in the three D-glucose concentrations. (**A**) Phase images of iPSC-derived myotubes at day 7 of differentiation. (**B**) Contraction traces of iPSC-derived myotubes derived from 5.55, 17.5 and 25 mM D-glucose myoblast cultures at day 7 of differentiation. (**C**) Quantification of fatigue indices of myotubes at day 7 of differentiation. Error bars = SEM. *P* ≤ 0.1, *; *P* ≤ 0.05, **; *P* ≤ 0.01, ***. *n* = 5 for low glucose and 3 for medium and high glucose. Statistics were performed utilizing One-way ANOVA with post-hoc Tukey HSD Test.

With the observed morphological differences between myotubes generated from hyperglycemic myoblasts and euglycemic myotubes (Figure 8A), an assessment was performed to delineate the functional implications of hyperglycemia. Myotubes from the euglycemic (5.55 mM) and hyperglycemic (17.5 mM and 25 mM) myoblast cultures were maintained for 7 days in differentiation and tested for relative myotube force using a previously described pixel subtraction system [15,18,19]. Myotubes from the three conditions were stimulated utilizing electrical field stimulation at 5V for 15 s, with frequencies of 0.33, 0.5, 1, 2 and 4 Hz. Comparatively, hyperglycemic myotubes demonstrated significantly increased excitation–contraction failure when the stimulation frequency reached 1 Hz and beyond (Figure 8B). Correspondent quantification of fatigue index indicated statistically higher fatigability at these high frequencies (1Hz, 2Hz and 4Hz) (Figure 8C). These results demonstrated that exposure of myoblasts to hyperglycemic conditions during their proliferative stage may affect eventual myofiber contraction function and, by extension, in vivo skeletal muscle function.

## 4. Discussion

Human skeletal muscle (hSKM) is the largest organ in the body, making up approximately 40% of the total human body weight and contains 50–70% of all body proteins [22]. Aside from its function in locomotion, posture maintenance and respiration, hSKM is also known as a primary site of glucose consumption and storage, and a reservoir for amino acids [23,24]. In recent times, the hSKM has also been reported to be an endocrine organ and a potent metabolic regulator [25]. The skeletal muscle secretes a plethora of myokines that systemically affect other systems including the liver, pancreas and immune system, among others [26,27]. Therefore, hSKM is critical for the maintenance of metabolic homeostasis and, as such, deleterious alterations in the hSKM have negative consequences on the overall health of an organism. This project aimed to study the specific effect of hyperglycemia on skeletal muscle generation, morphology and function without the confounding effects of other factors in the context of an investigation of diabetic myopathy pathogenesis. 

The initial observation was that myoblasts cultured in hyperglycemic conditions proliferated at a significantly slower pace compared to euglycemic conditions (Figure 1). This result is consistent with the studies utilizing mice DM models [11] and mice satellite cells [28]. Cell cycle analysis revealed a significant increase of cells in the S phase and G2/M phase under hyperglycemic conditions, suggesting retarded cell phase traversal. Similar results have been reported in human endothelial cell culture [29]. Meanwhile, elevated glucose can increase genomic instability and inhibit DNA repair [30,31]. However, high glucose has been found to be able to stimulate cell proliferation in endometrial cancer cell lines, the cell lines utilizing glucose as its primary energy source [32]. Presumably, in the iPSC-SKM culture, high glucose potentiated premature entry into cell proliferation, which generated DNA damage during replication, and then cell cycle arrest due to failure to pass the checkpoints. 

Glut4 is a key component in glucose homeostasis and the removal of glucose from circulation [33]. Glut4 downregulation is noted in diabetic myopathy [34]. However, acute hyperglycemia can induce an autoregulatory increase of Glut4 content in the plasma membrane of skeletal muscle cells through an insulin-independent mechanism, as demonstrated in C2C12 cells and rat skeletal muscle [35], although the contrary was also reported [36]. The glucose transport system can be upregulated in response to increasing levels of D-glucose, as observed in healthy individuals. Accordingly, increased Glut4 may suggest one mechanism to enhance the D-glucose uptake in response to hyperglycemic conditions. However, this remains inconclusive and would require further experimentation.

Among all known symptoms, hyperglycemia is specifically purported to cause metabolic and mitochondrial dysfunction in diabetic myopathy [2]. From our TMRE analysis, the inner mitochondrial membrane potential of myoblasts in the hyperglycemic conditions was shown to be significantly decreased, suggesting decreased efficiency in ATP production (Figure 6). The impairment of mitochondria function could contribute to all the cellular phenotypes observed, whether it was myoblast proliferation or myotube differentiation and function. Four mechanisms have been proposed as causes of hyperglycemia-induced mitochondrial dysfunction. They are the activation of the polyol pathway, hexosamine pathway induction, protein kinase C (PKC) activation and increased intracellular advanced glycation end-product formation [37,38]. While these hyperglycemia-induced conditions vary mechanistically, they all share increased reactive oxygen species (ROS) production as a common feature. ROS production is reportedly increased in hyperglycemic conditions and has been linked to decreased mitochondrial function [39,40,41]. Increased ROS concentration is believed to affect ATP production by uncoupling the inner mitochondrial membrane, which in turn disrupts the positive potential necessary to drive the electron transport chain [42]. Further investigations are needed to better understand the mechanisms of hyperglycemia-induced mitochondria deficiency. 

Hyperglycemic myoblasts in this study demonstrated an accelerated differentiation process compared to euglycemic myoblasts. This was evidenced by the relatively decreased MyoD intensity and Ki-67 positive cells in hyperglycemic myoblast cultures (Figure 2 and Figure 3), and a corresponding higher fusion index at early stages of these cultures once subjected to myotube formation (Figure 7). Downregulation of MyoD has been interpreted as a decline in myogenicity, which eventually results in impaired muscle regeneration. Accelerated fusion indicated a higher pool of myoblasts in the terminal differentiation-committed stage and a lower pool capable of self-renewal and proliferation, which in the long run would impair muscle mass maintenance and regeneration/repair. While myotubes differentiated from hyperglycemic myoblasts were comparatively larger, they were highly branched and had a generally disorganized MyHC structure (Figure 7). Correct arrangement of the contractile apparatus is vital to precise excitation–contraction coupling and function. Disorganization of the MyHC in the hyperglycemic myotubes could negatively affect contraction and force generation and sustainability, which was evidenced by the increased fatigue index among hyperglycemic myotubes compared to the control (Figure 8). High glucose-induced accelerated myogenesis has been reported to utilize a C2C12 cell line, which was proposed to be mediated by increased oxidative stress, and disorganization in hyperglycemic myotubes was reversed by reducing ROS levels as reported by Liu et al. [41]. The outcome of this research in addition to the findings of Liu and colleagues creates awareness of the implications of hyperglycemia-induced skeletal muscle dysfunction. Again, the human-based hyperglycemia model developed in this project would be beneficial in screening prospective therapeutics for alleviating hyperglycemia-induced sarcopenia.

While the clinical correlation of the observed branched morphology among the hyperglycemic myotubes is unclear, branching in skeletal muscle fibers could pose some discoordination in the contractile machinery that may affect function. The branch points can tentatively become stress points for tears in the tissue. Moreover, myofiber branching has been commonly found in muscular dystrophy and in regenerated or aged non-dystrophic muscles [43,44]. These branched myofibers are more susceptible to mechanical stress, and those found in the muscles of older individuals tend to be damaged irrevocably after eccentric contraction [45]. This study identified the myofiber branching pathology in the myoblast culture exposed to high glucose and thus revealed an important mechanism for the muscle mass loss and deficiency observed in hyperglycemia [9,46]. 

In this study, myoblasts were exposed to different levels of glucose only at the proliferation stage, yet the pathological phenotypes were identified not only for myoblasts, but also for the myotube fusion and myofiber functional stages. The lasting effect of brief high glucose exposure during myogenesis provokes concern about the detrimental effect of gestational hyperglycemia exposure to the health of the fetus in the situation of gestational diabetes mellitus (GDM). Although the extent of hyperglycemia exposure during the fetal stage has not been well characterized, children born to GDM mothers have higher body-mass-indices and even greater risk of developing type 2 diabetes very early in life [47,48]. Findings from this work and the clinical reports highlight the significance of blood sugar control during muscle development. The significant effect of high glucose observed on myogenesis and myofiber function and maintenance also raises questions for cell culture media that have glucose concentrations higher than the physiological level (5.55 mM).

Collectively, the cellular pathology of iPSC-myoblasts exposed to hyperglycemia conditions demonstrated a compromised regenerative capability as evidenced by reduced myoblast proliferation, impaired metabolism as evidenced by the reduced mitochondria efficiency, accelerated myotube fusion, increased formation of malformed (branched) myofibers, poor myofiber maintenance, and deficient function as evidenced by the increased contraction fatigue index. Interestingly, these myogenic phenotypes hold for both myoblasts derived from human iPSCs and adult-derived satellite cells (Supplementary Figures S1–S3), thus underscoring the importance of glucose regulation from gestation to adulthood. The phenotypes of decreased myoblast proliferation and deficient overall skeletal muscle regeneration are in line with studies conducted in both transgenic diabetic models and cell culture models [28,49,50], and those from clinical observation [51]. Diabetic muscle is reported to be much weaker and noted to demonstrate signs of impaired regeneration [9,52]. Among human subjects, exposure to hyperglycemia resulted in significant decreases in total muscle mass and skeletal muscle-associated mRNA levels [51]. By recapitulating these preclinical and clinical findings, this iPSC-SKM model provides a valid platform to study the pathogenesis of hyperglycemia. Further development of this human-based model to include other contributing factors of diabetic myopathy would be beneficial to gaining comprehensive understanding of the co-morbidity of diabetic myopathy. 

## 5. Conclusions

In summary, the outlined findings demonstrate the direct negative effect of hyperglycemia on human skeletal muscle regeneration and function, devoid of other diabetic complications. The cellular pathologies revealed concerning myoblast proliferation and later myotube differentiation and function provide important insights for the pathogenesis of diabetic myopathy. This defined human-based iPSC-derived skeletal muscle hyperglycemic model, with the flexibility and controllability for adding additional complexity, provides a valuable platform for mechanistic investigation of diabetic myopathy and therapeutic development.

## Data Availability

All the data for this work will be available upon proper request from the author.

## Supplementary Materials

The following supporting information can be downloaded at: https://www.mdpi.com/article/10.3390/cells11223674/s1, Figure S1: Effect of hyperglycemia on primary myoblast proliferation; Figure S2: Effect of hyperglycemia on cell cycle progression.; Figure S3: Phase contrast images of myotubes differentiated from adult-derived primary myoblasts previously expanded in varying D-glucose conditions concentration at days 3 and 6 of differentiation.
