# Supplementary material for: Hyperglycemia Negatively Affects IPSC-Derived Myoblast Proliferation and Skeletal Muscle Regeneration and Function
Source: Cells. 2022 Nov 18;11(22):3674. doi: 10.3390/cells11223674 (PMC9688533; doi:10.3390/cells11223674)
Supplement: Supplementary file 1 [file cells-11-03674-s001.zip › cells-1935985-supplementary.pdf]

Hyperglycemia Negatively Affects IPSC-Derived Myoblast Proliferation and Skeletal Muscle Regeneration and Function

Agnes Badu-Mensah[1, 2], Paola Valinski[2], Hemant Parsaud [1], James J Hickman[1, 2,*], Xiufang Guo[1, 2, *]

Supplementary Materials

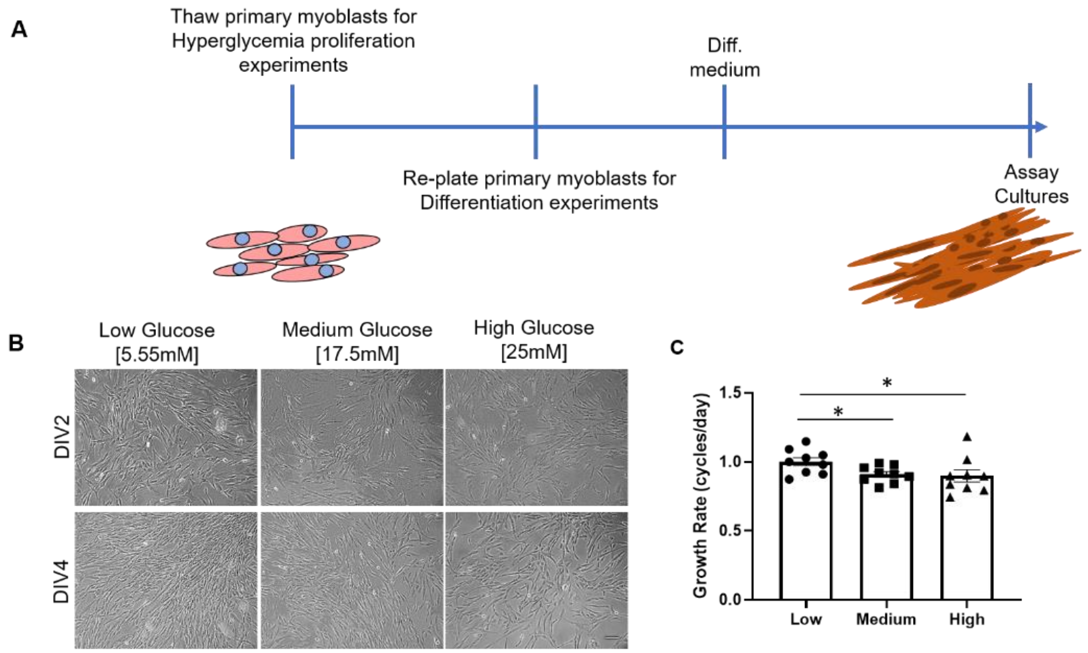

Figure S1. Effect of hyperglycemia on primary myoblast proliferation. **(A)** Illustration of primary myoblast culturing and differentiation timeline. **(B)** Phase images of primary myoblasts in 5.55 mM, 17.5 mM and 25 mM of glucose at days 2 and 4 of proliferation. **(C)** Graph showing primary myoblasts proliferation rate in varying glucose concentration. Scale bar = 100 µm Error bars = SEM. $P \leq 0.1$, *; $P \leq 0.05$, **; $P \leq 0.01$, ***. N=3. Statistics was performed utilizing One-way ANOVA with post-hoc Tukey HSD Test by comparing between each hyperglycemia group with the euglycemic control group.

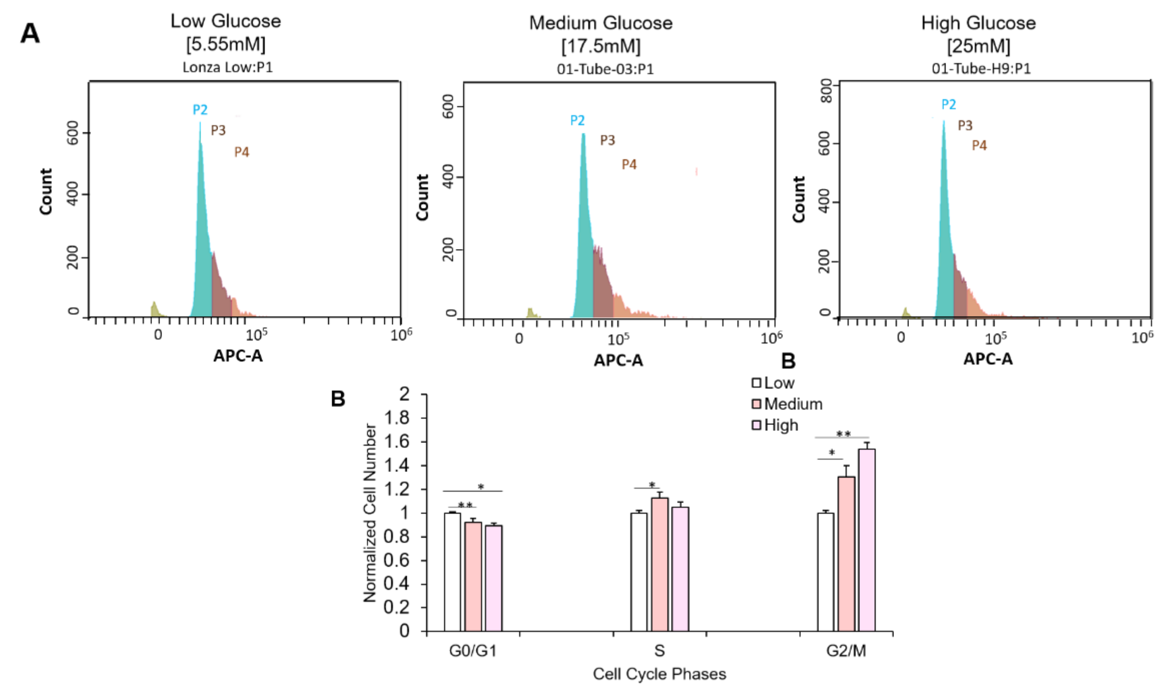

Figure S2. Effect of hyperglycemia on cell cycle progression. **(A)** Pictograph showing primary myoblasts distribution among the various cell cycle phase in each glucose concentration (P2, green: G0/G1 phase; P3, dark brown, S phase; P4, light brown, G2/M phase). **(B)** Quantification of primary myoblasts at the various phases of cell cycle in 5.55 mM, 17.5 mM and 25 mM of D-glucose conditions. Data was normalized to the cell count of each phase at euglycemic condition. Error bars = SEM. $P \leq 0.1$, *; $P \leq 0.05$, **; $P \leq 0.01$, ***. N=10. Statistics was performed utilizing One-way ANOVA with post-hoc Tukey HSD Test.

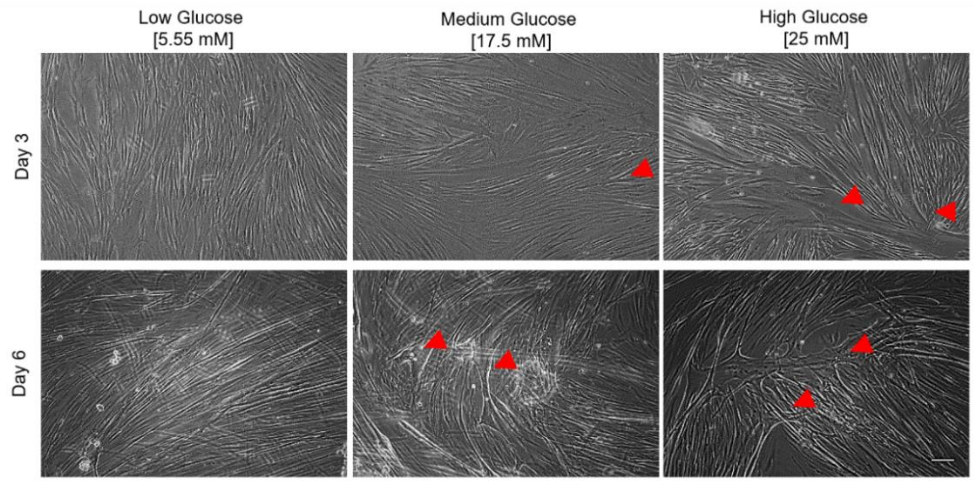

Figure S3. Phase contrast images of myotubes differentiated from adult-derived primary myoblasts previously expanded in varying D-glucose conditions concentration at days 3 and 6 of differentiation. Scale bar = 100 µm. Red arrows pointing to branched hypertrophic myotubes.
